# Icariin, but Not Genistein, Exerts Osteogenic and Anti-apoptotic Effects in Osteoblastic Cells by Selective Activation of Non-genomic ERα Signaling

**DOI:** 10.3389/fphar.2018.00474

**Published:** 2018-05-11

**Authors:** Ming-Xian Ho, Christina C.-W. Poon, Ka-Chun Wong, Zuo-Cheng Qiu, Man-Sau Wong

**Affiliations:** ^1^Department of Applied Biology and Chemical Technology, The Hong Kong Polytechnic University, Kowloon, Hong Kong; ^2^Institute of Traditional Chinese Medicine and Natural Products, College of Pharmacy, Jinan University, Guangzhou, China; ^3^State Key Laboratory of Chinese Medicine and Molecular Pharmacology (Incubation), The Hong Kong Polytechnic University Shenzhen Research Institute, Shenzhen, China; ^4^Shenzhen Key Laboratory of Food Biological Safety Control, The Hong Kong Polytechnic University Shenzhen Research Institute, Shenzhen, China

**Keywords:** phytoestrogens, osteoblasts, apoptosis, MAPK/ERK, PI3K/Akt

## Abstract

Genistein and icariin are flavonoid compounds that exhibit estrogen-like properties in inducing bone formation and reducing bone loss associated with estrogen deficiency in both preclinical and clinical studies. However, the mechanisms that are involved in mediating their estrogenic actions in bone cells are far from clear. The present study aimed to study the signaling pathways that mediate the estrogenic actions of genistein and icariin in osteoblastic cells. The effects of genistein and icariin on the activation of estrogen receptor (ER) and the downstream mitogen-activated protein kinase (MAPK) and phosphatidylinositol 3-kinase (PI3K)/Akt signaling pathway in murine osteoblastic MC3T3-E1 cells and rat osteoblastic UMR-106 cells were studied. As expected, genistein displayed higher binding affinity toward ERβ than ERα and significantly induced estrogen response element (ERE)-dependent transcription in UMR-106 cells in a dose-dependent manner. In contrast, icariin failed to bind to ERα or ERβ and did not induce ERE-dependent transcription in UMR-106 cells at 10^-10^ to 10^-7^ M. The effects of genistein (10 nM) and icariin (0.1 μM) on cell proliferation and differentiation in osteoblastic UMR-106 cells were abolished in the presence of ER antagonist ICI 182,780 (1 μM), MAPK inhibitor U0126 (10 μM), and PI3K inhibitor LY294002 (10 μM). Genistein at 10 nM rapidly induced ERK1/2 phosphorylation at 5–10 min in UMR-106 cells and the phosphorylation of ERα at both Ser118 and Ser167 in both MC3T3-E1 and transfected UMR-106 cells whereas icariin at 0.1 μM rapidly activated both ERK1/2 and Akt phosphorylation in UMR-106 cells and subsequent ERα phosphorylation at both Ser118 and Ser167 in MC3T3-E1 and transfected UMR-106 cells. Confocal imaging studies confirmed that the phosphorylation of ERα at Ser 118 and Ser 167 by genistein and icariin in MC3T3-E1 cells was mediated via MAPK- and PI3K-dependent pathway, respectively. Furthermore, our studies showed that icariin exerted stronger anti-apoptotic effects than genistein and 17β-estradiol (E2) and inhibited the cleavage of downstream caspase-3 in MC3T3-E1 cells induced by a potent PI3K inhibitor, PI828 (at 2 μM). These results indicated that the mechanisms that mediate the estrogenic actions of icariin in osteoblastic cells are different from those of genistein.

## Introduction

Concern for the use of hormone replacement therapy (HRT) in increasing postmenopausal women’s risk of breast cancer, stroke, thrombosis, and cardiovascular diseases (Rossouw et al., 2002; Beral and Million Women Study Collaborators, 2003) has triggered research efforts to develop alternative approaches for managing menopausal syndromes and preventing postmenopausal osteoporosis. Phytoestrogens are compounds present in plants with estrogen-like biological activities and different phytoestrogen preparations are popular among postmenopausal women as dietary supplement for management of menopausal symptoms (Lagari and Levis, 2010; Gil-Izquierdo et al., 2012) and they might be useful for management of bone health (Weaver and Cheong, 2005; Poulsen and Kruger, 2008; Zhang et al., 2008a; Lagari and Levis, 2014).

Flavonoid compounds, especially soy isoflavones, are by far the most frequently studied phytoestrogens that have been reported (Weaver and Cheong, 2005; Poulsen and Kruger, 2008; Zhang et al., 2008a,b; Lagari and Levis, 2010, 2014). Genistein has been shown in some studies to exert estrogen-like bone protective effects to OVX rats and mice (Zhang et al., 2008a) and enhance osteoblastic differentiation and maturation as well as inhibit osteoclast formation (Poulsen and Kruger, 2008). Several observational studies reported that high dietary soy intake among Asian populations was associated with higher bone mineral density (BMD) and lower incidence of osteoporosis-related fractures (Lagari and Levis, 2010). However, results from clinical studies on the BMD of Western women tested with soy foods or isolated soy isoflavones are inconsistent (Weaver and Cheong, 2005; Zhang et al., 2008a; Lagari and Levis, 2010). Furthermore, as the *in vivo* effects of phytoestrogens are similar to the effects of estrogens and their actions are mediated through ERs (ERα and ERβ), there are increasing safety concerns over the effect of long-term exposure to phytoestrogens (Bedell et al., 2014). With the recent increase in the number of study and application of diverse types of phytoestrogens, it is of prime importance to understand the mechanism of actions of each type of phytoestrogen for better prediction of their therapeutic profiles and for avoiding their potential adverse side effects upon long-term exposure.

It is well known that both genomic and non-genomic ER signaling pathways can mediate estrogenic actions. In the classical genomic pathway, ERs are activated by directly binding to estrogens, which alters gene transcription via interacting with EREs in the promoters of target genes (Cheskis et al., 2007). In addition, estrogen induces responses that are very rapid (measured in seconds to minutes) and independent of transcriptional events (Levin and Hammes, 2016). Such rapid non-genomic responses are mediated by extra-nuclear ER and require unique post-translational modifications and protein–protein interactions of the receptor with adaptor molecules, G proteins, and kinase (Banerjee et al., 2014). In non-genomic signaling pathway, estrogen can initiate membrane signaling through growth factor receptors or membrane-associated ER, an initiation that subsequently leads to the activation of ER by phosphorylation via extracellular regulated kinase/MAPK (ERK/MAPK) or phosphatidyl-inositol-3-kinase/AKT (PI3K/AKT) in a ligand-independent manner (Likhite et al., 2006). Indeed, the anti-apoptotic actions of estrogens in osteoblasts were shown to be mediated by the extra-nuclear ER signaling via the activation of Src/Shc/ERK pathway (Kousteni et al., 2003; Almeida et al., 2006) and ER–ERK–mTOR pathway (Yang et al., 2013). These studies suggested that kinase initiated actions of estrogens via ERK and PI3K/AKT played an important role in mediating the non-reproductive actions of estrogens.

Icariin, an 8-prenylated flavonoid glucoside, is the bioactive compound (Mok et al., 2010; Liang et al., 2012; Ming et al., 2013) that accounts for the osteoprotective effects of the commonly prescribed Chinese herb *Herba Epimedii* (HEP; Xie et al., 2005; Zhu et al., 2012) and its flavonoid extract (Chen et al., 2011). Our previous study indicated that icariin (Mok et al., 2010) and the total flavonoid fraction of HEP (Chen et al., 2011) could protect against bone loss and bone deterioration associated with estrogen deficiency in mice without exerting any uterotrophic effects. A study by Zhang et al. (2007) also demonstrated that a preparation containing 60 mg icariin, 15 mg daidzein, and 3 mg genistein could reduce bone loss in late postmenopausal women in a 24-month randomized, double-blind, and placebo-controlled trial. The *in vitro* osteogenic and anti-resorptive effects of icariin on bone mesenchymal stem cells, osteoblasts, and osteoblast-like cells as well as on bone marrow stromal cells and RAW264.7 cells have been extensively reported (Xu et al., 2010; Cao et al., 2012; Li et al., 2015; Luo et al., 2015). Most importantly, icariin has been demonstrated to have stronger osteogenic activity than genistein in rat calvarial osteoblasts due to its prenyl group on C-8 as the active group (Ma et al., 2011). Our previous studies showed that icariin, unlike genistein, did not stimulate ERα- or ERβ-mediated ERE-dependent luciferase activities, but was able to rapidly induce ERα phosphorylation at Ser118 in UMR-106 cells (Mok et al., 2010). These studies suggest that icariin is a unique class of phytoestrogens that exert bone-specific effects via rapid ER signaling pathways in bone cells. However, it is unclear if icariin and genistein would mimic estrogen in exerting anti-apoptotic effects in osteoblasts via the activation of rapid ER signaling pathways. Icariin has been newly reported to promote the maturation and mineralization of primary cilia of rat calvarial osteoblasts by activating the cAMP/protein kinase A (PKA)/cAMP response element-binding protein (CREB) pathway (Shi et al., 2017). This new evidence suggests that the action of icariin in osteoblastic cells might also involve G protein-coupled signaling pathway.

The present study is designed to study the bone anabolic effects of icariin and genistein as well as to determine if they exert any anti-apoptotic effects in osteoblastic cells via rapid ER signaling pathways. It is hoped that our study can provide new insights into understanding the complex biological actions of different phytoestrogens in bone cells.

## Materials and Methods

### Materials

17β-Estradiol (E2; purity ≥ 98%) and genistein (GEN; purity ≥ 98%) were purchased from Sigma-Aldrich (St. Louis, MO, United States). Icariin (ICA; purity ≥ 98%) was purchased from LKT Laboratories Inc (St. Paul, MN, United States). ICI 182,780 (purity > 99%), U0126 (purity > 99%), LY294002 (purity > 99%), and PI828 (purity > 99%) were purchased from TOCRIS Bioscience (Bristol, United Kingdom). Minimum essential medium α (α-MEM), Dulbecco’s modified Eagle medium (DMEM), fetal bovine serum (FBS), charcoal-stripped fetal bovine serum (sFBS), trypsin, and penicillin-streptomycin were the products of Gibco (Life Technologies, Rockville, MD, United States). Antibodies against phospho-ERK1/2 (Tyr204), ERα, phospho-ERα (Ser118), phosphor-Akt, and Akt were purchased from Santa Cruz Biotechnology (Santa Cruz, CA, United States). Antibody against ERK-1/2, phospho-Akt (Ser473) and caspase-3 were purchased from Cell Signaling Technology (Beverly, MA, United States). Antibody against phospho-ERα (Ser167) and acridine orange (AO; 10 mg/ml in water) were purchased from Molecular Probes (Life Technologies, Rockford, IL, United States). Antibody against β-actin was purchased from Abcam (Cambridge, United Kingdom). Plasmids encoding ER-α, ER-β, and pERE-luc were kindly provided by Dr. Vincent Giguere (McGill University, Montreal, QC, Canada).

### Culture of Murine MC3T3-E1 Cells and Rat UMR-106 Cells

The murine pre-osteoblastic MC3T3-E1 cells (Subclone 4, CRL-2593, ATCC) and rat osteoblast-like UMR-106 cells (CRL-1661, ATCC) were maintained in α-MEM and DMEM, respectively, and supplemented with 10% FBS and antibiotics (100 units/ml penicillin and 100 μg/ml streptomycin). Cells were incubated at 37°C in an atmosphere of 95% air humidity and 5% CO_2_. The cells were trypsinized with phenol red-free 0.05% trypsin-EDTA (Gibco, United States) and sub-cultured when they reached 80–90% confluence. For induction of cell differentiation of MC3T3-E1 cells into mature osteoblastic cells, culture medium was supplemented with 10 mM β-glycerophosphate and 50 μg/ml ascorbic acid as osteogenic medium. To provide steroid-free condition in MC3T3-E1 cells and UMR-106 cells, culture media were replaced with hormone-depleted media (phenol red-free α-MEM and DMEM supplemented with 5 and 1% sFBS, respectively) for 24 h prior to all drug treatments (E2 or GEN or ICA or antagonists). For cell proliferation assay, osteoblastic cells were treated with different dosages of genistein and icariin (10^-5^–10^-12^ M), 17β-estradiol (10^-8^ M), and vehicle for 48 h. The cell proliferation was examined by using colorimetric measurement – MTS (3-(4,5-dimethylthiazol-2-yl)- 5- (3-carboxy-methoxyphenyl)-2-(4-sulfophenyl)-2H-tetrazolium) assay (Promega, Ann Arbor, MI, United States) as previously described (Mok et al., 2010). The absorbance at 490 nm was measured on a microplate reader (Bio-Rad Laboratories, Inc., United States). For alkaline phosphatase (ALP assay), osteoblastic cells were treated with vehicle, 17β-estradiol (10^-8^ M), genistein (10^-5^–10^-12^ M), or icariin (10^-5^–10^-12^ M) for 7 days. The cell lysates were then collected to determine the ALP activities using a LabAssay ALP Kit (Wako, Osaka, Japan) following the manufacturer’s instruction as previously described (Mok et al., 2010). The absorbance at 405 nm was measured on a microplate reader (Bio-Rad laboratories, Inc., United States). ALP activity of each sample was normalized with its total protein content determined by Bradford protein assay and expressed as units per milligram of protein. To determine the role of ER, MAPK, and PI3K/Akt signaling in the actions of flavonoids, UMR-106 cells transfected with 0.4 μg ER-α plasmid were pre-treated with specific ER inhibitor ICI 182,780 (ICI, 1 μM), ERK1/2 inhibitor U0126 (10 μM), and PI3K inhibitor LY294002 (10 μM) for 24 h prior to drug treatments (E2: 10 nM or GEN: 10 nM or ICA: 0.1 μM; 48 h). Their effects on cell proliferation and ALP activities were determined as described. For the blocking experiments, the dosages of genistein and icariin were chosen based on our previous studies (Chen and Wong, 2006; Mok et al., 2010; Xiao et al., 2014) in which the most effective dosages of genistein and icariin in inducing osteoblastic cell proliferation and differentiation under steroid-free culture condition were 10 nM and 0.1 μM, respectively.

### Competitive Radioligand Binding Assay With Purified ERα and ERβ

Competitive radioligand binding assay with purified human ERα and ERβ was performed as described in our previous study (Xiao et al., 2014). Briefly, seven dilutions (10^-11^–10^-5^ M) of GEN and ICA were examined. The radioactivity of each sample was detected by a liquid scintillation counter (Beckman LS6500, United States) and was expressed as disintegration per minute (dpm). The binding of ^3^[H]E2 to ERα or ERβ in the presence of competitor was determined by subtracting the non-specific binding and expressed as the percentage of total binding without competitor. The percentages of relative binding affinity (RBA) were calculated by (IC_50_17β-estradiol/IC_50_ compound) × 100.

### Transient Transfection and ER-Mediated ERE Transcription Activity Assay

UMR-106 cells were transfected with 0.4 μg ER-α or ER-β plasmid, 0.4 μg pERE-luc, and 0.1 μg pRL-TK (internal control reporter plasmid) using Lipofectamine 2000 reagents (Invitrogen, United States) for 6 h, and then treated with E2 (10^-8^ M), GEN (10^-10^–10^-6^ M), or ICA (10^-10^–10^-6^ M) or vehicle for 24 h. Upon treatment, lyzed cells were collected and luciferase activities were measured using the Dual Luciferase Reporter Assay System (Promega, Ann Arbor, MI, United States). Luminescent signals were detected by Glomax-20/20 Luminometer (Promega, Ann Arbor, MI, United States). The estrogen responsive ERE transcriptional activity was expressed as *firely* luciferase values normalized to pRL-TK *Renilla* luciferase values.

### Immunoblotting

Treated osteoblastic cell lysates were collected using lysis buffer [20 mM Tris-HCl, 150 mM NaCl, 5 mM EDTA, 67 mM sodium pyrophosphate, 0.5 mM sodium orthovanadate, and 1% Triton X-100 (vol./vol.); pH 7.5] supplemented with protease inhibitors (2 μg/ml aprotinin, 2 μg/ml leupeptin, and 1 mM PMSF). Protein concentrations were measured using bicinchoninic acid (BCA) assay (Thermo Fisher Scientific, Rockford, IL, United States). Equal amounts of proteins (50 μg per lane) were separated on 10 or 12% SDS-polyacrylamide gels and electro-transferred onto PVDF membranes (Millipore, Danvers, MA, United States). Protein expressions of phospho-ERα (Ser118)/ERα, phospho-ERα (Ser167)/ERα, p-MEK/MEK, p-ERK/ERK, p-Akt/Akt, pro-caspase-3, and cleaved caspase-3 were determined by using the appropriate primary antibodies (**Table 1**). β-actin was used as control for equal protein loading. The membranes were washed and immunoblotted with anti-rabbit IgG (1:1000; Millipore, Danvers, MA, United States) or anti-mouse IgG (1:1000; Cell Signaling Technology, Beverly, MA, United States) or anti-goat IgG (1:1000; Thermo Fisher Scientific, Rockford, IL, United States) conjugated with horseradish peroxidase. SuperSignal^®^ West Pico chemiluminescent substrate (Thermo Fisher Scientific, Rockford, IL, United States) was used to detect the labeled protein bands. The chemiluminescence intensity of each band was measured by Azure c600 imaging system (Azure Biosystems, Dublin, CA, United States) and quantified using ImageJ program (NIH, Bethesda, MD, United States).

**Table 1:** The primary antibodies used in this study.

Name of Antibody	Manufacturer Details	Dilution Used	Species Raised
phospho-ERα (Ser118)	Santa Cruz #sc-12915	1:500 for Western blotting 1:200 for immunostaining	Goat
phospho-ERα (Ser167)	Thermo Fisher Scientific #PA5-37570	1:1000 for Western blotting 1:200 for immunostaining	Rabbit
ERα (C-3)	Santa Cruz #sc-514857	1:500 for Western blotting 1:200 for immunostaining	Mouse
Phospho-ERK1/2 (Tyr204)	Santa Cruz #sc-7383	1:500	Mouse
ERK1/2 (p44/42 MAPK)	Cell Signaling Technology #9102	1:1000	Rabbit
Phospho-Akt (Ser473)	Cell Signaling Technology #4058	1:1000	Rabbit
Caspase-3	Cell Signaling Technology #9662	1:1000	Rabbit
β-Actin	Abcam #ab6276	1:2000	Mouse

### Acridine Orange Staining for Apoptosis and Confocal Imaging

MC3T3-E1 cells (cell density of 1 × 10^-3^ cells/ml as single cell layer) were seeded on 20 mm × 20 mm glass coverslips (Marienfeld, Germany) and cultured overnight. The cells were then treated with either vehicle (control), E2 (10 nM), GEN (10 nM, 0.1 μM), or ICA (10 nM, 0.1 μM) for 1, 6, and 24 h and/or PI828 (2 μM; 24 h) pre-treatment in steroid-free condition. The anti-apoptotic functions of genstein and icariin in osteoblastic cells under steroid-free culture condition were evaluated at 10 nM and 0.1 μM, concentrations that are likely to be achieved in the human circulation. Treated cells were washed with pre-warmed PBS and incubated with 1 μg/ml AO dye at 37°C for 15 min as described in Hu et al. (2015). AO-stained cells were fixed immediately with 4% paraformaldehyde and mounted on glass slides for scanning. Fluorescence images were captured at mid-plane of cells (oil objective; magnification: 400X) by a Leica TCS SPE DMi8 confocal microscope (Leica Microsystems, Wetzlar, Germany). AO excitation was 458 nm, emission was 480–660 nm (from green to yellow to red fluorescence), indicating different stages of apoptosis (Mpoke and Wolfe, 1997). The overall intensities of the fluorescent signals of single cell were also quantified using the corresponding Leica Microsystems software station (LAS AF, Leica Microsystems, Germany).

### Immunostaining

Treated MC3T3-E1 cells were fixed immediately with 4% paraformaldehyde and were permeabilized with 0.5% Triton X-100 for 1 min. Upon blocking with 1% BSA for 45 min, the cells were incubated with specific primary antibodies [phospho-ERα (Ser118) or phospho-ERα (Ser167) and ERα; concentration 1:200] overnight at 4°C. The primary antibodies were probed with either Alexa Fluor 488-conjugated anti-rabbit antibody or Alexa Fluor 594-conjugated anti-mouse antibody for 1 h at room temperature. DAPI counter-staining was applied for determining the intensity of single cell. Fluorescence images were captured at mid-plane of cells (oil objective; magnification: 600X) by the Leica TCS SPE DMi8 confocal microscope (Leica Microsystems, Wetzlar, Germany). The overall intensities of the fluorescent signals of single cell were also quantified using the corresponding Leica Microsystems software station (LAS AF, Leica Microsystems, Germany).

### Data and Statistical Analysis

All quantitative data were presented as mean ± SEM and analyzed by GraphPad PRISM^®^ 5.0 software (San Diego, CA, United States). Comparisons between groups were performed using either unpaired Student’s *t*-test or one-way ANOVA followed by Tukey’s post-tests, where appropriate. *p* < 0.05 was considered significant.

## Results

### Genistein and Icariin Stimulated Osteoblastic Cell Proliferation and Differentiation

To determine the dose (10^-12^–10^-5^ M)-dependent effects of genistein and icariin on osteoblastic functions, their effects on cell proliferation and ALP activities in murine pre-osteoblastic MC3T3-E1 and rat osteoblast-like UMR-106 cells were studied. 17β-estradiol (E2) at 10^-8^ M significantly stimulated MC3T3-E1 (**Supplementary Figure S1A**) and UMR-106 (**Supplementary Figure S1C**) cell proliferation (*p* < 0.05 vs. control). Similarly, genistein significantly stimulated MC3T3-E1 cell proliferation at 10^-9^–10^-5^ M (*p* < 0.05, **Supplementary Figure S1A**) and UMR-106 cell proliferation at a lower concentration (10^-9^ M) but inhibited UMR-106 cell proliferation at 10^-5^ M (**Supplementary Figure S1C**). Icariin significantly increased MC3T3-E1 cell proliferation at 10^-8^–10^-5^ M (*p* < 0.05, **Supplementary Figure S1A**) while stimulated UMR-106 cell proliferation at 10^-9^–10^-6^ M (*p* < 0.05, **Supplementary Figure S1C**). E2 at 10^-8^ M significantly stimulated MC3T3-E1 (**Supplementary Figure S1B**) and UMR-106 (**Supplementary Figure S1D**) ALP activities (*p* < 0.01 vs. control). Similarly, genistein significantly increased ALP activities at 10^-7^ and 10^-6^ M in MC3T3-E1 cells (*p* < 0.05, **Supplementary Figure S1B**) and at low concentrations (10^-11^–10^-9^ M) in UMR-106 cells (*p* < 0.05, **Supplementary Figure S1D**). Icariin significantly increased ALP activities at all tested concentrations in MC3T3-E1 cells (*p* < 0.05, **Supplementary Figure S1B**) and in UMR-106 cells (*p* < 0.05, **Supplementary Figure S1D**). The results indicated that icariin exerted bone anabolic effects in both types of osteoblastic cells at concentrations as low as 10^-12^ M while higher concentrations of genistein (10^-9^–10^-6^ M) are required to induce anabolic effects in MC3T3-E1 cells.

### Genistein, but Not Icariin, Exhibited Specific Binding to ERα and ERβ and Induced ERE-Dependent Gene Transcription in UMR-106 Cells

**FIGURE 1:**
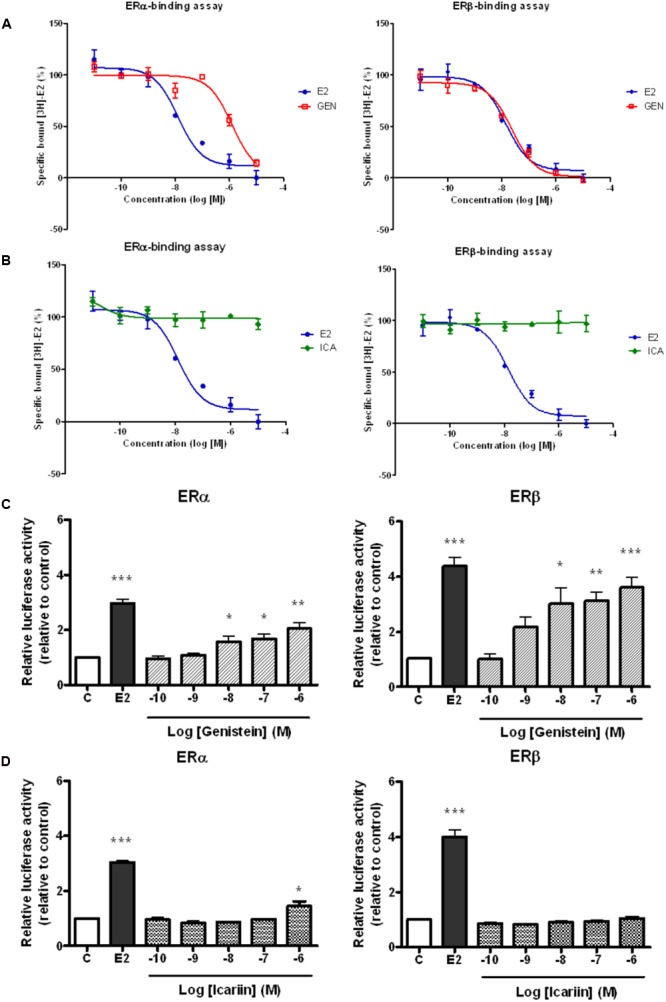
Competitive binding curves of 17β-estradiol (E2, 10 nM), **(A)** genistein (GEN; 10 nM), and **(B)** icariin (ICA; 0.1 μM) to ERα and ERβ. The binding of [^3^H]E2 to recombinant human ERα and ERβ was determined by subtracting the disintegration per minute (dpm) from the non-specific binding. The percentages of specific bound [^3^H]E2 were expressed as mean ± SEM (*n* = 5). Effects of treatment with **(C)** genistein and **(D)** icariin on ERα- or ERβ-mediated ERE luciferase activity in transfected UMR-106 cells. The transfected UMR-106 cells were treated with vehicle (C), 17β-estradiol (E2, 10^-8^ M), genistein (10^-10^–10^-6^ M), or icariin (10^-10^–10^-6^ M) for 24 h. Firely and Renilla luciferase activity was measured sequentially with Dual Luciferase Assay reagents. Results were expressed as mean ± SEM. ^∗^*p* < 0.05, ^∗∗^*p* < 0.01, and ^∗∗∗^*p* < 0.001 versus the control (*n* = 5).

It is well known that genistein is a phytoestrogen that binds specifically to both ERα and ERβ. To determine if the actions of icariin also involve direct binding with ERs, the binding affinities of icariin compared with genistein toward purified human ERα and ERβ were examined by competitive radiolabeled binding assay. **Figure 1** demonstrates the competition binding curves of genistein (**Figure 1A**) and icariin (**Figure 1B**) to ERα and ERβ in comparison to those of E2. The IC_50_ value of 17β-estradiol for ERα was 1.26 × 10^-8^ M and it was 1.39 × 10^-8^ M for ERβ. These values are close to the theoretical value of 1 × 10^-8^ M, confirming the validity of the assay. The results revealed that increasing concentration of genistein (10^-11^–10^-5^ M), but not icariin, displaced the binding of [^3^H]-E2 to both ERα and ERβ (IC_50_ for ERα = 1.27 × 10^-6^ M; ERβ = 2.39 × 10^-8^ M). The results also confirmed that genistein exhibited a greater binding affinity to ERβ (RBA = 58.1%) than to ERα (RBA = 0.99%). Most importantly, icariin failed to bind to either ERα or ERβ specifically at concentrations up to 10^-5^ M. As shown in **Figure 1C**, genistein (10^-10^–10^-6^ M) significantly increased ERE-luciferase activities in a dose-dependent manner in UMR-106 cells via both ERα and ERβ. In addition, the ability of genistein to promote ERE-luciferase transcription via ERβ was apparently greater than via ERα. Icariin at most tested concentrations failed to induce ERE-luciferase activity in UMR-106 cells co-transfected with ERα or ERβ (**Figure 1D**). Icariin in high dosage (1 μM) significantly induced a modest increase (1.4-fold) in ERα-mediated ERE transcription in UMR-106 cells. These results indicated that icariin at most of effective concentration for inducing anabolic effects in osteoblastic cells did not involve the activation of the classical ER signaling pathway.

### ER, MAPK, and PI3K Antagonists Abolished the Stimulatory Effects of Genistein and Icariin on Cell Proliferation and ALP Activities of Transfected UMR-106 Cells

**FIGURE 2:**
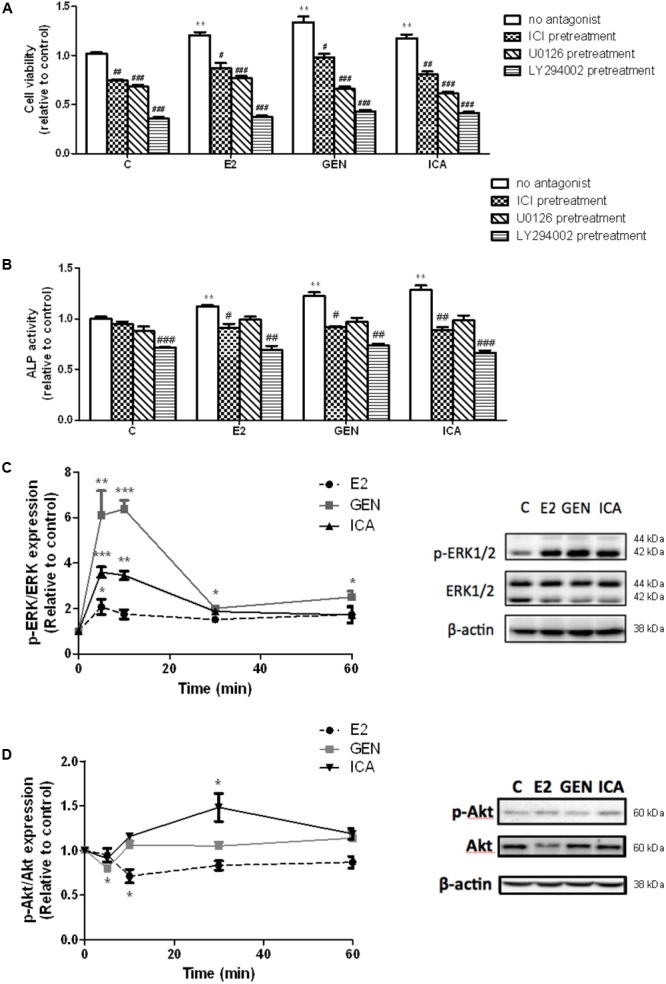
The effects of antagonists on flavonoid-induced proliferation and differentiation in the transfected UMR-106 cells. The transfected cells were pre-treated with specific ER inhibitor ICI 182,780 (ICI, 1 μM), MEK inhibitor U0126 (10 μM), and PI3K inhibitor LY294002 (10 μM) for 24 h prior to drug treatments (E2: 10 nM or GEN: 10 nM or ICA: 0.1 μM; 48 h) or vehicle. **(A)** Cell proliferation was assessed by cell viability MTS assay and **(B)** cell differentiation was assessed by ALP activity kit. Results were expressed as mean ± SEM. ^∗∗^*p* < 0.01 versus the control. ^#^*p* < 0.05, ^##^*p* < 0.01, and ^###^*p* < 0.001 versus the same treatment group without antagonists (*n* = 4). The time-course effects of genistein and icariin on phosphorylation of **(C)** ERK1/2 and **(D)** Akt in transfected UMR-106 cells. Cells were treated with vehicle, 17β-estradiol (E2; 10 nM), genistein (GEN; 10 nM), or icariin (ICA; 0.1 μM) for 5, 10, 30, and 60 min. Representative immunoblots showing the protein expressions of p-ERK1/2, ERK1/2, p-Akt, and Akt at 10 min in transfected UMR-106 cells. The phosphorylation levels of ERK1/2 and Akt were determined by immunoblotting and expressed as mean ± SEM. ^∗^*p* < 0.05, ^∗∗^*p* < 0.01, and ^∗∗∗^*p* < 0.001 versus the control (*n* = 3).

To determine if rapid signaling pathways are involved in mediating the estrogen-like anabolic effects of genistein and icariin in osteoblasts, transfected UMR-106 cells were pre-treated with specific ER antagonist ICI182,780, ERK antagonist U0126, and PI3K antagonist LY294002 before treatment with E2 (10 nM), genistein (10 nM), and icariin (0.1 μM). Cells pre-treated with ICI 182,780 (1 μM), U0126 (10 μM), and LY294002 (10 μM) completely abolished the stimulatory effects of E2 (10 nM), icariin (0.1 μM), and genistein (10 nM) on cell proliferation (**Figure 2A**), while the cells only pre-treated with ICI 182,780 and LY294002, but not U0126, significantly reduced ALP activities induced by E2, icariin, and genistein in transfected UMR-106 cells (**Figure 2B**). The results showed that the effects of genistein and icariin on osteoblastic cell proliferation were ER-, ERK/MAPK-, and PI3K/Akt-dependent and their effects on differentiation were ER- and PI3K/Akt-dependent.

### The Responses of ERK and Akt Phosphorylation to Treatment With Genistein and Icariin in Transfected UMR-106 Cells

Genistein (10 nM) and icariin (0.1 μM) significantly increased the phosphorylation of ERK1/2, but not MEK (**Supplementary Figure S2**), in transfected UMR-106 cells within 10 min of incubation (**Figure 2C**). The time-dependent effects of genistein and icariin on ERK signaling in transfected UMR-106 cells were then investigated. As shown in **Figure 2C**, E2 at 10 nM slightly increased ERK1/2 phosphorylation in transfected UMR-106 cells in 5 min (2.1-fold; *p* < 0.05 vs. control) and sustained the action within an hour of incubation. Similarly, icariin significantly increased ERK1/2 phosphorylation to a greater magnitude in 5 min (3.6-fold; *p* < 0.05 vs. control) in transfected UMR-106 cells whereas genistein markedly induced rapid phosphorylation of ERK1/2 in 5 min (6.1-fold; *p* < 0.01 vs. control) and reached the maximum at 10 min to result in a 6.4-fold increase in pERK/ERK ratio in transfected UMR-106 cells (*p* < 0.001 vs. control; **Figure 2C**). Icariin (0.1 μM), but not genistein (10 nM), induced Akt phosphorylation in transfected UMR-106 cells upon treatment for 30 min (**Figure 2D**). The time-dependent effects of genistein and icariin on Akt phosphorylation in transfected UMR-106 cells were then studied. Treatment of transfected UMR-106 cells with E2 gradually reduced the basal Akt phosphorylation within 10 min by 0.7-fold (*p* < 0.05 vs. control) and the level of phosphorylation gradually returned to the basal level by 30 min of incubation (**Figure 2D**). Treatment with genistein sharply reduced the basal Akt phosphorylation in transfected UMR-106 cells within 5 min by 0.8-fold (*p* < 0.05 vs. control) and the level of phosphorylation returned to the basal level by 10 min of incubation (**Figure 2D**). In contrast, icariin significantly increased Akt phosphorylation in transfected UMR-106 cells by 1.5-fold (*p* < 0.05 vs. control) upon treatment for 30 min and the phosphorylation level gradually returned to the basal level by 60 min of incubation (**Figure 2D**). These results indicated that only icariin could induce Akt phosphorylation in transfected UMR-106 cells.

### Genistein and Icariin Induced Rapid ERα Phosphorylation at Ser118 and Ser167 in Osteoblastic MC3T3-E1 Cells and Transfected UMR-106 Cells

**FIGURE 3:**
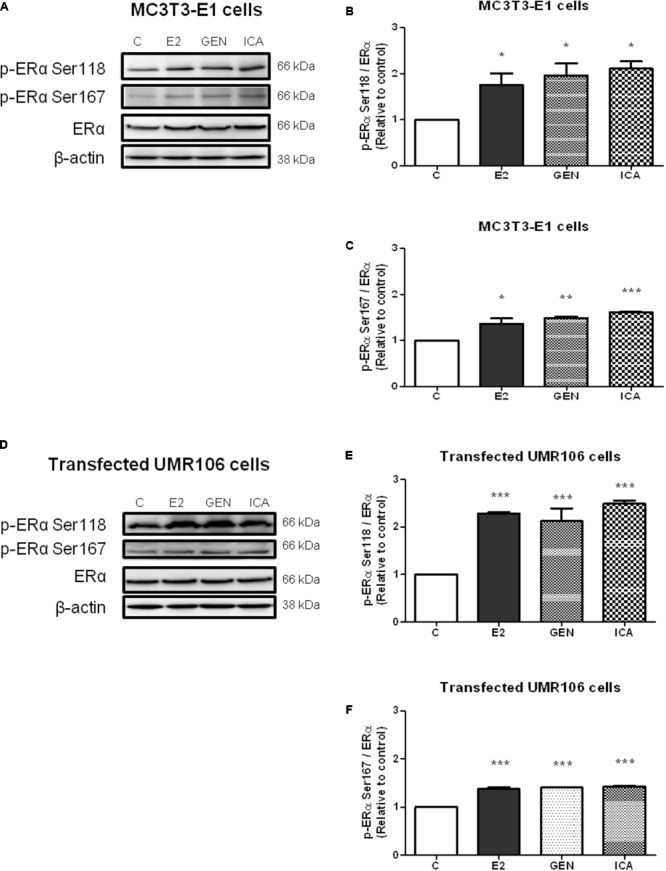
The acute effects of genistein and icariin on phosphorylation of ERα (Ser118) and ERα (Ser167) in MC3T3-E1 cells and UMR-106 cells. Cells were treated with vehicle (C), 17β-estradiol (E2; 10 nM), genistein (GEN; 10 nM), or icariin (ICA; 0.1 μM) for 10 min. Representative immunoblots showing the protein expressions of p-ERα (Ser118), p-ERα (Ser167), ERα, and β-actin **(A)** in MC3T3-E1 cells and **(D)** transfected UMR-106 cells. The ratio of p-ERα (Ser118)/ERα protein expression in **(B)** MC3T3-E1 cells and **(E)** transfected UMR-106 cells and the ratio of p-ERα (Ser167)/ERα protein expression in **(C)** MC3T3-E1 cells and **(F)** transfected UMR-106 cells were shown. Results were expressed as mean ± SEM. ^∗^*p* < 0.05, ^∗∗^*p* < 0.01, and ^∗∗∗^*p* < 0.001 versus the control (*n* = 3).

**FIGURE 4:**
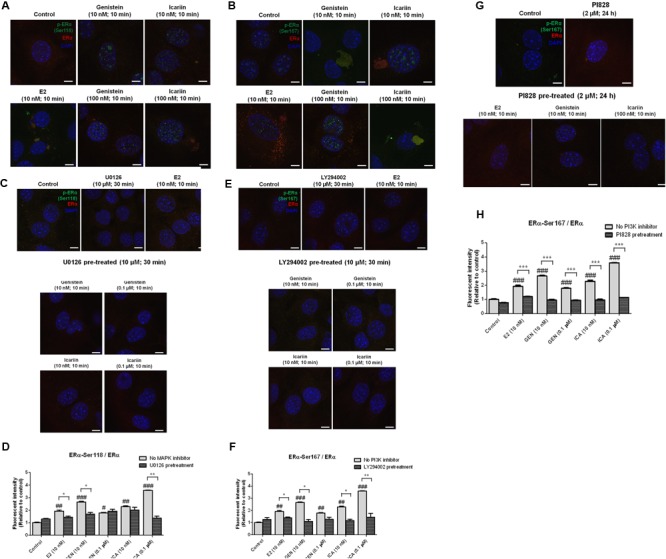
The rapid stimulatory effects of genistein and icariin on ERα phosphorylation at Ser118 and Ser167 were abolished in osteoblastic MC3T3-E1 cells by the pre-treatments of MAPK inhibitor (U0126) and PI3K inhibitors (LY294002 and PI828). Representative fluorescence images of flavonoid-induced cellular co-localization of **(A)** p-ERα Ser118 and ERα, **(B)** p-ERα Ser167 and ERα, **(C)** p-ERα Ser118 and ERα in the presence of U0126, **(E)** p-ERα Ser167 and ERα in the presence of LY294002, and **(G)** p-ERα Ser167 and ERα in the presence of PI828 in osteoblastic MC3T3-E1 cells. The images were captured at mid-plane of cells and visualized using confocal laser scanning microscope (magnification: 600X) and the total fluorescent intensities were measured using fluorescent Alexa Fluor 488-conjugated antibodies labeled p-ERα Ser167 (shown as green) and Alexa Fluor 594-conjugated antibodies labeled ERα (shown as red). DAPI counter-staining (blue) was applied for determination of single cell. Cells were treated with vehicle (control), 17β-estradiol (E2; 10 nM), genistein (10 nM, 0.1 μM), or icariin (10 nM, 0.1 μM) for 10 min with or without pre-treatment of U0126 (10 μM; 30 min), LY294002 (10 μM; 30 min), or PI828 (2 μM; 24 h). The total fluorescent intensities of **(D)** p-ERα Ser118/ERα and **(F,H)** p-ERα Ser167/ERα in each single cell were quantified and expressed as mean ± SEM. ^∗^*p* < 0.05, ^∗∗^*p* < 0.01, ^∗∗∗^*p* < 0.001 and ^#^*p* < 0.05, ^##^*p* < 0.01, ^###^*p* < 0.001 versus the control. Scale bar: 5 μm (*n* = 5).

To examine if genistein and icariin could induce rapid ERα phosphorylation at Ser118 and Ser 167 in osteoblastic cells, the relative ratio of p-ERα (Ser118) and p-ERα (Ser167) to ERα expression in MC3T3-E1 cells (**Figures 3A–C**) and UMR-106 cells (**Figures 3D–F**) in response to treatment for 10 min was examined by immunoblotting. E2 (10 nM), genistein (10 nM), and icariin (0.1 μM) significantly increased both protein expression levels of p-ERα (Ser118)/ERα (**Figures 3A,B**) and p-ERα (Ser167)/ERα (**Figures 3B,C**) in MC3T3-E1 cells within 10 min of treatment. Similar responses were also shown in transfected UMR-106 cells (*p* < 0.001; **Figures 3D–F**). The rapid stimulation of ERα phosphorylation at Ser118 and at Ser167 in MC3T3-E1 osteoblastic cells by genistein and icariin was also confirmed by confocal microscopy. As shown in **Figure 4A**, genistein and icariin at 10 nM and 0.1 μM induced ERα phosphorylation at Ser118 (which appeared as green) in MC3T3-E1 cells within 10 min of incubation and the induction appeared to be close to the nucleus (which stained in blue by DAPI). Similar responses of ER phosphorylation at Ser167 to treatment with genistein and icariin are shown in **Figure 4B**.

### Genistein and Icariin Induced ERα Phosphorylation at Ser118 in Osteoblastic Cells via MAPK-Dependent Pathway

To determine if MAPK-dependent pathway was involved in the activation of ERα phosphorylation at Serine 118 by genistein and icariin, MC3T3-E1 cells were pre-treated with MAPK inhibitor U0126 (10 μM) for 30 min, followed by treatment with genistein and icariin for 10 min. Pre-treatment with U0126 completely reduced the intensity of green fluorescent signals (p-ERα at Ser118) in MC3T3-E1 cells treated with genistein and icariin at both 10 nM and 0.1 μM (**Figure 4C**). The expression levels of p-ERα (Ser118)/ERα were also quantified as the fluorescent intensities. Our results clearly indicated that E2 at 10 nM, and genistein and icariin at 10 nM and 0.1 μM significantly increased fluorescent intensities (p-ERα (Ser118)/ ERα) in MC3T3-E1 cells, with the highest induction found in cells treated with 0.1 μM of icariin (**Figure 4D**). Pre-treatment with U0126 significantly reduced the induction of fluorescent intensities in MC3T3-E1 cells treated with 0.1 μM of icariin, 10 nM of genistein, and 10 nM of E2. Pre-treatment of UMR-106 cells with U0126 (10 μM) also abolished the stimulatory effects of genistein and icariin on ERα phosphorylation at Ser118 (**Supplementary Figure S3A**).

### Genistein and Icariin Induced ERα Phosphorylation at Ser167 in Osteoblastic Cells via PI3K-Dependent Pathway

To determine if PI3K-dependent pathway was involved in the activation of ERα phosphorylation at Serine 167 by genistein and icariin, MC3T3-E1 cells were pre-treated with PI3K inhibitor LY294002 (10 μM) for 30 min, followed by treatment with genistein and icariin for 10 min. As shown in **Figure 4E**, pre-treatment with LY294002 significantly reduced the intensity of green fluorescent signals (p-ERα at Ser167) in MC3T3-E1 cells treated with genistein and icariin at both 10 nM and 0.1 μM. Our results clearly indicated that all treatment groups had significantly increased fluorescent intensities [p-ERα (Ser167)/ERα] in MC3T3-E1 cells, with the highest induction found in cells treated with 0.1 μM of icariin (**Figure 4F**). Pre-treatment with LY294002 significantly reduced the induction of fluorescent intensities [p-ERα (Ser167)/ERα] in MC3T3-E1 cells treated with 10 nM to 0.1 μM of icariin, 10 nM of genistein, and 10 nM of E2. Pre-treatment of UMR-106 cells LY294002 (10 μM) also abolished the stimulatory effects of genistein and icariin on ERα phosphorylation at Ser167 (**Supplementary Figure S3B**). The effects of genistein (10 nM, 0.1 μM) and icariin (10 nM, 0.1 μM) on rapid ERα phosphorylation at Ser167 and the involvement of PI3K in osteoblastic MC3T3-E1 cells were further investigated by using a potent PI3K inhibitor (PI828 at 2 μM). The stimulatory effects of E2, genistein, and icariin on p-ERα (Ser167)/ERα were completely abolished in MC3T3-E1 cells by pre-treatment with PI828 for 24 h (*p* < 0.001; **Figures 4G,H**). These results confirmed that the PI3K pathway was involved in mediating the rapid estrogenic actions of genistein and icariin.

### Icariin Had Stronger Anti-apoptotic Effects Than E2 and Genistein on Suppressing PI828-Induced Apoptosis in MC3T3-E1 Cells

**FIGURE 5:**
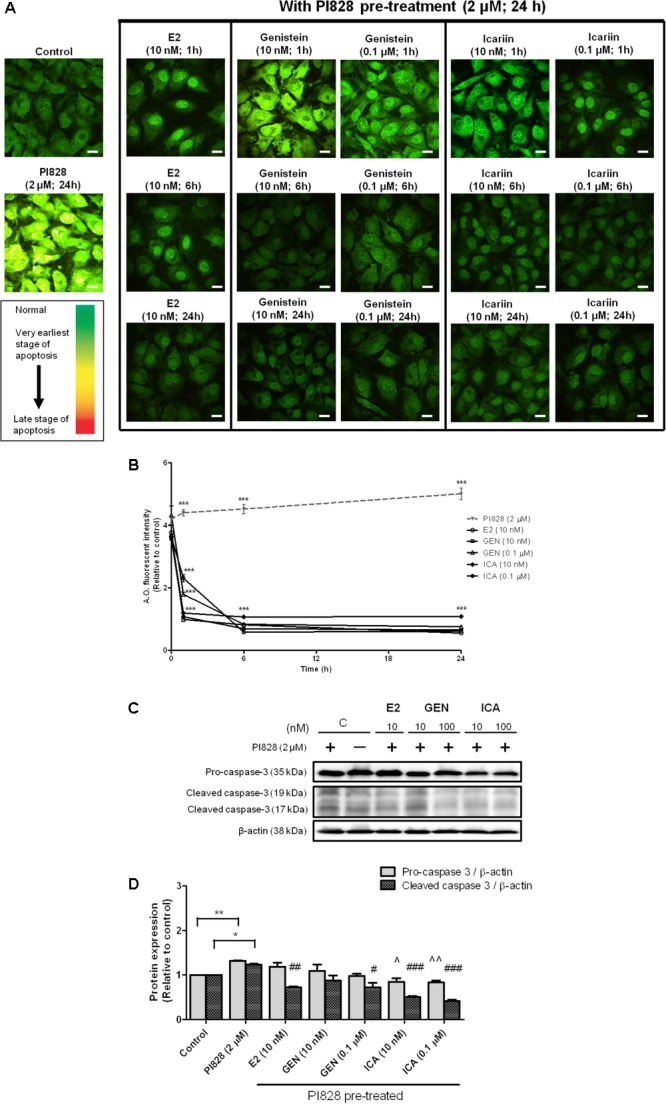
The anti-apoptotic effects of 17β-estradiol (E2), genistein, and icariin on PI3K inhibitor-induced apoptosis in MC3T3-E1 cells. Cells were pre-treated with PI828 (2 μM; 24 h) followed by the treatment of 17β-estradiol (E2; 10 nM), genistein (10 nM, 0.1 μM), icariin (10 nM, 0.1 μM), or vehicle (control) for 1, 6, and 24 h. **(A)** Representative fluorescence images of acridine orange (AO) staining in MC3T3-E1 cells. AO-stained cells in green fluorescence indicated cells with normal nuclei randomly distributed, yellow fluorescence indicated cells with condensed nuclei at the early stage of apoptosis, and orange-red fluorescence indicated cells with highly condensed and apoptotic nuclei at the late stage of nuclear death. The images were captured at mid-plane of cells and visualized using confocal laser*scanning microscope (magnification: 400X). **(B)** The total fluorescent intensity of single cell after PI828 pre-treatment at 1, 6, and 24 h was quantified and expressed as mean ± SEM. ^∗∗∗^*p* < 0.001 versus the PI828 treatment group. Scale bar: 10 μm (*n* = 10). The anti-apoptotic effects of 17β-estradiol (E2), genistein, and icariin on protein expressions of pro-caspase-3 and cleaved caspase-3 in osteoblastic MC3T3-E1 cells. Cells were treated with vehicle (C), 17β-estradiol (E2; 10 nM), genistein (GEN; 10 nM), or icariin (ICA; 0.1 μM) for 1 h with or with PI828 pre-treatment (2 μM; 24 h). **(C)** Representative immunoblots showing the protein expressions of pro-caspase-3 and cleaved caspase-3 in osteoblastic MC3T3-E1 cells. **(D)** The ratio of pro-caspase-3/β-actin and cleaved caspase-3/β-actin protein expressions in MC3T3-E1 cells were expressed as mean ± SEM. ^∗^*p* < 0.05, ^∗∗^*p* < 0.01, ˆ*p* < 0.05, ˆˆ*p* < 0.01, and ^#^*p* < 0.05, ^##^*p* < 0.01, ^###^*p* < 0.001 versus the corresponding PI828 treatment group (*n* = 3).*

To determine if genistein and icariin could protect osteoblastic cells against apoptosis, MC3T3-E1 cells were pre-treated with PI828 to induce apoptosis for 24 h prior to treatment with 10 nM or 0.1 μM of genistein and icariin for 1–24 h. Different stages of apoptosis in cells can be monitored by confocal microscopy by using AO staining. Green fluorescence indicated the cells with normal nuclei randomly distributed, yellow fluorescence indicated the cells with condensed nuclei at early stage of apoptosis, and orange-red fluorescence indicated the cells with highly condensed and apoptotic nuclei at late stage of nuclear death (Mpoke and Wolfe, 1997). **Figure 5A** illustrates that bright yellow-green fluorescence in cytoplasm with yellow-orange apoptotic nuclei was found in MC3T3-E1 cells in response to pre-treatment with PI828. Treatments of MC3T3-E1 cells with E2, genistein, and icariin significantly suppressed the PI828-induced apoptosis in time-dependent manner (*p* < 0.001; **Figure 5B**). Most importantly, icariin (0.1 μM) exhibited stronger anti-apoptotic effects than genistein as demonstrated by its superior ability to suppress PI828-induced apoptosis in MC3T3-E1 cells within 1 h of treatment (*p* < 0.001; **Figures 5A,B**). Caspase-3 is a protease that plays a central role in the execution-phase of cell apoptosis (Elmore, 2007). To further determine if genistein and icariin could protect against apoptosis in osteoblastic cells by suppressing caspase-3 activity, their effects on protein expression of pro-caspase-3 and cleaved caspase-3 in MC3T3-E1 pre-treated with PI828 were determined. As shown in **Figures 5C,D**, PI828 significantly increased pro-caspase-3 and cleaved caspase-3 protein expression in MC3T3-E1 cells (*p* < 0.01). Treatment of MC3T3-E1 cells with E2 at 10 nM, genistein and icariin at 10 nM and 0.1 μM for 10 min significantly suppressed PI828-induced caspase-3 activation (cleaved caspase-3 expression; *p* < 0.05, **Figure 5D**). Most importantly, icariin, but not E2 nor genistein, could significantly reduce the pro-caspase-3 expression levels in osteoblastic MC3T3-E1 cells (*p* < 0.05, **Figure 5D**). These results clearly demonstrated that icariin could exert anti-apoptotic effects in osteoblastic cells by suppressing caspase-3 cleavage.

## Discussion

Previous studies by us and others clearly demonstrated that icariin (Zhao et al., 2008; Mok et al., 2010; Pang et al., 2010; Song et al., 2013; Liao et al., 2014; Xiao et al., 2014; Zhou et al., 2014; Zhang et al., 2016) and genistein (Heim et al., 2004; Chen and Wong, 2006; Morris et al., 2006; Zhang et al., 2007) exerted estrogen-like protective effects to bone *in vitro* and *in vivo*. However, the signaling mechanisms by which these two flavonoids exert estrogenic actions, particularly in osteoblasts, are not fully understood. The present study systematically characterized the effects of icariin and genistein on inducing classical (genomic) and extra-nuclear (non-genomic) estrogen signaling cascades in osteoblastic cells. Most importantly, our study demonstrated that the estrogenic actions of icariin and genistein were mediated by different mechanisms of actions. Icariin exerted osteogenic and anti-apoptotic actions in osteoblastic cells by selective activation of non-genomic estrogen signaling cascades, while the actions of genistein were mediated by activation of both genomic and non-genomic estrogen signaling.

The actions of phytoestrogen are traditionally believed to be mediated by ERs via classical genomic pathways in which phytoestrogens, such as genistein, bind to ERα and ERβ and induce ERE-dependent transcriptional activities in target tissues. Our results confirmed previous observations (Kuiper et al., 1997; Morito et al., 2001) that genistein showed greater binding affinity to ERβ than ERα, and induced ERE-dependent transcription at 100-fold lower concentration via ERβ than ERα in transfected UMR-106 cells. In contrast, icariin did not exhibit any specific binding to either ERα or ERβ nor induce ERE-dependent transcription at 10^-10^–10^-6^ M in transfected UMR-106 cells. Thus, icariin can be regarded as a non-binder by definition (Matthews et al., 2000) as it failed to displace more than 10% of the radioactive [^3^H]E2 in binding to ERs even at concentration 1000-fold higher than the latter. The lack of binding of icariin to ERs could be due to steric hindrance of the side groups in glycoside that prevented its interaction with the binding domain of the receptors (Morito et al., 2001). These results suggest that the actions of icariin, unlike genistein, were not mediated via classical genomic estrogen signaling.

The role of ERα in mediating the osteogenic effects in bone has been well documented (Khalid and Krum, 2016). Apart from classical genomic pathway, ERα can be activated ligand-independently by a variety of extracellular signals, including growth factors [such as epidermal growth factor (EGF) and insulin-like growth factor-1 (IGF-1)], by making use of the rapid cellular signaling cascades including MAPK/ERK signaling (Kato et al., 1995; Bunone et al., 1996; Chen et al., 2002) and PI3K/Akt signaling (Campbell et al., 2001; Likhite et al., 2006). Using pathway-specific blockers, our study showed that the osteogenic effects of genistein (10 nM) and icariin (0.1 μM) at their most effective dosages were ER-dependent and involved the activation of MAPK/ERK signaling for cell proliferation and PI3K/Akt signaling for cell differentiation. The induction of MAPK/ERK signaling in osteoblastic cells by both genistein and icariin was rapid (within 5–10 min of incubation) and was similar to the reported non-genomic actions of E2 in bone cells (Kousteni et al., 2001). Indeed, the involvement of ERK and ER in the osteogenic actions of icariin in MC3T3-E1 cells was also reported by others (Song et al., 2013). Akt is the downstream signaling protein that mediates PI3K-initiated signaling in the regulation of cell cycle and cell survival (Datta et al., 1999; Jeong et al., 2008). Our study indicated that Akt phosphorylation induced by icariin in UMR-106 cells occurred by 30 min of incubation and its phosphorylation was even suppressed by treatment with genistein and estrogen. The decrease in Akt phosphorylation by E2 in UMR-106 cells was also reported by others (Sunters et al., 2010), and in the same study, IGF-1 was shown to induce Akt phosphorylation in these cells. Thus, our results indicate that the actions of icariin in activating PI3K/Akt signaling pathways in osteoblastic cells appear to be different from those of genistein and estrogen.

Our study demonstrated that both genistein and icariin could induce rapid phosphorylation of ERα at Ser 118 and Ser 167 in osteoblastic cells, as revealed by immunoblotting and immunostaining using confocal microscopy. Most importantly, such rapid phosphorylation could be abolished by co-treatment with an MAPK blocker (U0126) and PI3K blockers (LY294002 and PI828), thus confirming the role of non-genomic signaling pathways in mediating the estrogenic actions of genistein and icariin in osteoblastic cells. Such “extra-nuclear” or “membrane-initiated” estrogen signaling pathways (Levin and Hammes, 2016; Yaşar et al., 2016), which take place within seconds to minutes, mediate transcription-independent effects of ERs and modulate the actions of nuclear ERs, transcription factors, other regulatory proteins as well as chromatin complex via post-translational modifications. Upon exposure to E2, these rapid post-translational modifications trigger ER translocation to the cell membrane, re-distribution, as well as association with other signaling proteins, thereby exerting rapid estrogenic actions (Yaşar et al., 2016). The recruitment of ER to the cell membrane will facilitate its interaction with various G-proteins to generate rapid signals such as cAMP and cGMP and initiate the stimulation of kinase cascades, including MAPK/ERK and PI3K/AKT pathways (Pedram et al., 2002; Levin, 2018). In fact, the rapid ER signaling in breast cancer epithelium also requires the transactivation of EGFR and/or IGFR1 (Hammes and Levin, 2007). Thus, our results indicate that both genistein and icariin could trigger these rapid signaling events in osteoblastic cells. However, as icariin, unlike genistein, is unable to trigger ligand-dependent activation of ER, its estrogenic actions in osteoblastic cells are likely to be mediated solely by non-genomic signaling pathways.

Anti-apoptotic actions of estrogens in osteoblasts were previously shown to be mediated by extra-nuclear ER signaling (Kousteni et al., 2003; Almeida et al., 2006; Yang et al., 2013). Apoptosis is a natural process in adult skeleton that contributes to physiological bone turnover, repair, and regeneration (Bran et al., 2008). Osteoblast apoptosis is believed to be the third most common cause that contributes to the pathological conditions in osteoporosis. The loss of bone mass and strength due to aging is associated with the increase in the prevalence of apoptosis among osteoblasts and osteocytes, which result in a decrease in osteoblast number and rate of bone formation (Hughes and Boyce, 1997; Almeida et al., 2007; Liu et al., 2007). Thus, osteoblast apoptosis could be an important target for the prevention of osteoporosis. Our study clearly showed that genistein and icariin mimicked the effects of E2 in suppressing PI3K inhibitor-induced apoptosis in MC3T3-E1 cells and such effects can be accounted for by their abilities to trigger rapid extra-nuclear ER signaling. Moreover, as revealed by confocal microscopy and caspase-3 activities, our results demonstrated that icariin has stronger anti-apoptotic effects than genistein and E2 in osteoblastic cells. Such activity of icariin might be due to its anti-oxidative properties as reported by Ma et al. (2014) who found that icariin (0.1 μM) could significantly reduce oxidative stress by decreasing reactive oxygen species (ROS) and malondialdehyde production and increasing superoxide dismutase activity, thereby arresting cell cycle in hypoxic rat calvarial osteoblasts. However, the ability of icariin to stimulate Akt phosphorylation as demonstrated in the present study might also account for its stronger anti-apoptotic effects in osteoblastic cells as Akt is known to regulate apoptosis post-translationally and transcriptionally (Datta et al., 1999; Neri et al., 2002). Nevertheless, these results further confirmed the importance of extra-nuclear signaling pathways in mediating the anti-apoptotic effects of flavonoids in osteoblastic cells.

Some previous studies reported that icariin exerted higher osteogenic potency than genistein (Ma et al., 2011, 2014; Wang et al., 2018) and suggested that the presence of the 8-prenyl group in icariin might account for such effects. The present study further demonstrated that the actions of icariin were distinct from those of genistein and E2 in which icariin selectively activated non-genomic signaling pathways and induced Akt phosphorylation in osteoblastic cells. However, it is unclear if this unique mechanism of action of icariin could account for its higher osteogenic activities than genistein in osteoblastic cells. Moreover, it would be of interest to determine if the presence of 8-prenyl group contributes to the unique abilities of icariin to activate Akt phosphorylation in bone cells. Indeed, an earlier study in our laboratory also reported that prenylation at C-8 position of genistein could increase its osteogenic activity (Zhang et al., 2008b). Future study will be needed to further delineate the biological targets and/or signaling pathways by which icariin or flavonoids containing 8-prenyl group elicits stronger osteogenic and anti-apoptotic effects in bone cells.

**FIGURE 6:**
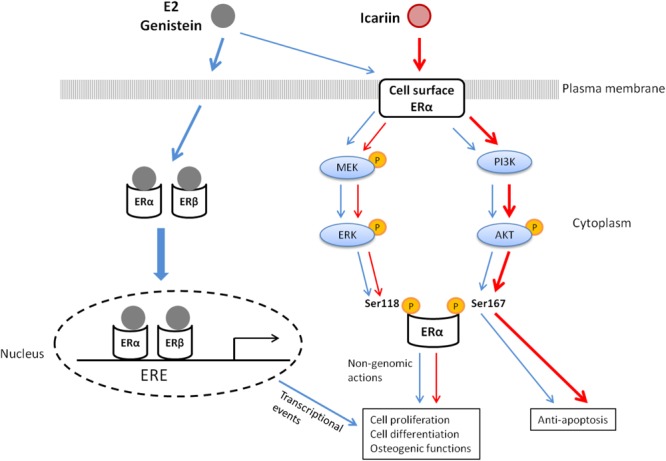
The proposed mechanisms of action of 17β-estradiol (E2), genistein, and icariin in osteoblastic cells.

**Figure 6** summarizes the potential mechanisms by which genistein and icariin exert bone anabolic and anti-apoptotic effects. These include: (1) genistein mimics the actions of estrogen in inducing ERE-dependent transcriptional activities and triggers rapid non-genomic signaling via the stimulation of MAPK/ERK and PI3K/Akt pathways and the subsequent phosphorylation of ERα in osteoblastic cells; (2) icariin selectively induces non-genomic signaling via the activation of MAPK/ERK and PI3K/Akt pathways, and the subsequent phosphorylation of ERα in osteoblastic cells. As icariin does not bind to ERs, the cellular targets that directly interact with icariin to elicit non-genomic signaling in osteoblastic cells remain unknown. As growth factor (such as IGF-1) plays an important role in bone growth and remodeling (Guntur and Rosen, 2013; Tian et al., 2018) and its receptor (IGF1R) is an upstream regulator of both MAPK and PI3K signaling pathways (Fuentes et al., 2011), it is possible that IGF-1 and IGF1R might play a role in mediating the actions of icariin in osteoblastic cells. In particular, just like icariin, IGF-1 is known to induce Akt phosphorylation in osteoblastic cells (Sunters et al., 2010). Future study will be needed to determine if IGF-IR/IGF-1 is involved in mediating the actions of icariin and other flavonoids in bone cells.

The present study was performed in two osteoblastic cell lines, namely, UMR-106 and MC3T3-E1 cells. UMR-106 cell line was chosen as a model in our study as it was a rat osteosarcoma cell with more mature osteoblastic phenotypes that were commonly used by others for studying the effects of different hormonal agents (PTH, prostaglandins, and bone resorbing steroids) on signal transduction (Partridge et al., 1983; Forrest et al., 1985; Mitchell and Goltzman, 1990; Armbrecht et al., 1998). MC3T3-E1 Subclone 4 cell line was chosen as it was a murine pre-osteoblastic cell that exhibited high levels of osteoblast differentiation upon culturing in the presence of ascorbic acid and 3–4 mM inorganic phosphate and it was believed to behave with properties very similar to primary calvarial osteoblasts (Wang et al., 1999). Due to the concern of yield and reproducibility of using primary osteoblasts for signal transduction study, primary cells such as bone marrow stromal cells or primary calvarial osteoblasts have not been employed in the present study. Future study will be needed to validate the effects of icariin and genistein in triggering rapid ER and their anti-apoptotic effects using primary osteoblastic cells.

## Conclusion

Our results confirm that genistein and icariin are phytoestrogens that exert estrogenic actions in osteoblastic cells. Most importantly, the mechanisms that mediate the estrogenic action of icariin are different from those of genistein. The unique ability of icariin to selectively activate non-genomic estrogen signaling, and in particular, Akt phosphorylation, might account for its superior osteogenic and anti-apoptotic actions in osteoblastic cells. Its potential application as non-hormonal therapy for improving osteogenic functions for menopausal women, without inducing any undesirable estrogen-dependent transcriptional events, is worth further investigation.

## Author Contributions

M-XH and CP performed the experiments, analyzed the data, prepared the figures, and drafted the manuscript. K-CW and Z-CQ helped analyzing data. M-SW designed the study.

## Conflict of Interest Statement

The authors declare that the research was conducted in the absence of any commercial or financial relationships that could be construed as a potential conflict of interest.

## Supplementary Material

FIGURE S1 The stimulatory effects of genistein and icariin on cell proliferation and differentiation in **(A,B)** MC3T3-E1 cells and **(C,D)** UMR-106 cells. Cells were treated with vehicle (C), 17β-estradiol (E2, 10^-8^M), icariin (10^-12^–10^-5^ M), and genistein (10^-12^–10^-5^ M) for cell proliferation MTS assay and ALP activity in MC3T3-E1 cells and UMR-106 cells after 48 h and 7 days, respectively. Results were expressed as mean ± SEM. ^∗^*p* < 0.05, ^∗∗^*p* < 0.01, and ^∗∗∗^*p* < 0.001 versus the control (*n* = 5).

FIGURE S2 Representative immunoblots showing the protein expressions of p-MEK1/2 and MEK1/2 in transfected UMR-106 cells. Cells were treated with vehicle (C), 17β-estradiol (E2, 10^-8^ M), genistein (GEN; 10 nM), or icariin (ICA; 0.1 μM) for 10 min (*n* = 3).

FIGURE S3 The inhibitory effects of the pre-treatment of **(A)** MAPK inhibitor (U0126) and **(B)** PI3K inhibitor (LY294002) on flavonoids-induced ERα phosphorylation at Ser118 and Ser167 in osteoblastic MC3T3-E1 cells. Cells were treated with vehicle, genistein (GEN; 10 nM), or icariin (ICA; 0.1 μM) for 10 min after pre-treatment of U0126 (10 μM; 30 min) or LY294002 (10 μM; 30 min). Representative immunoblots showing the protein expressions of p-ERα (Ser118), p-ERα (Ser167), ERα, and β-actin in MC3T3-E1 cells (*n* = 3).

## Abbreviations

ER: estrogen receptor
ERE: estrogen response element
MAPK: mitogen-activated protein kinase
PI3K: phosphatidylinositol 3-kinase
