# Supplementary figures and images for: Icariin, but Not Genistein, Exerts Osteogenic and Anti-apoptotic Effects in Osteoblastic Cells by Selective Activation of Non-genomic ERα Signaling
Source: Front Pharmacol. 2018 May 11;9:474. doi: 10.3389/fphar.2018.00474 (PMC5958194; doi:10.3389/fphar.2018.00474)

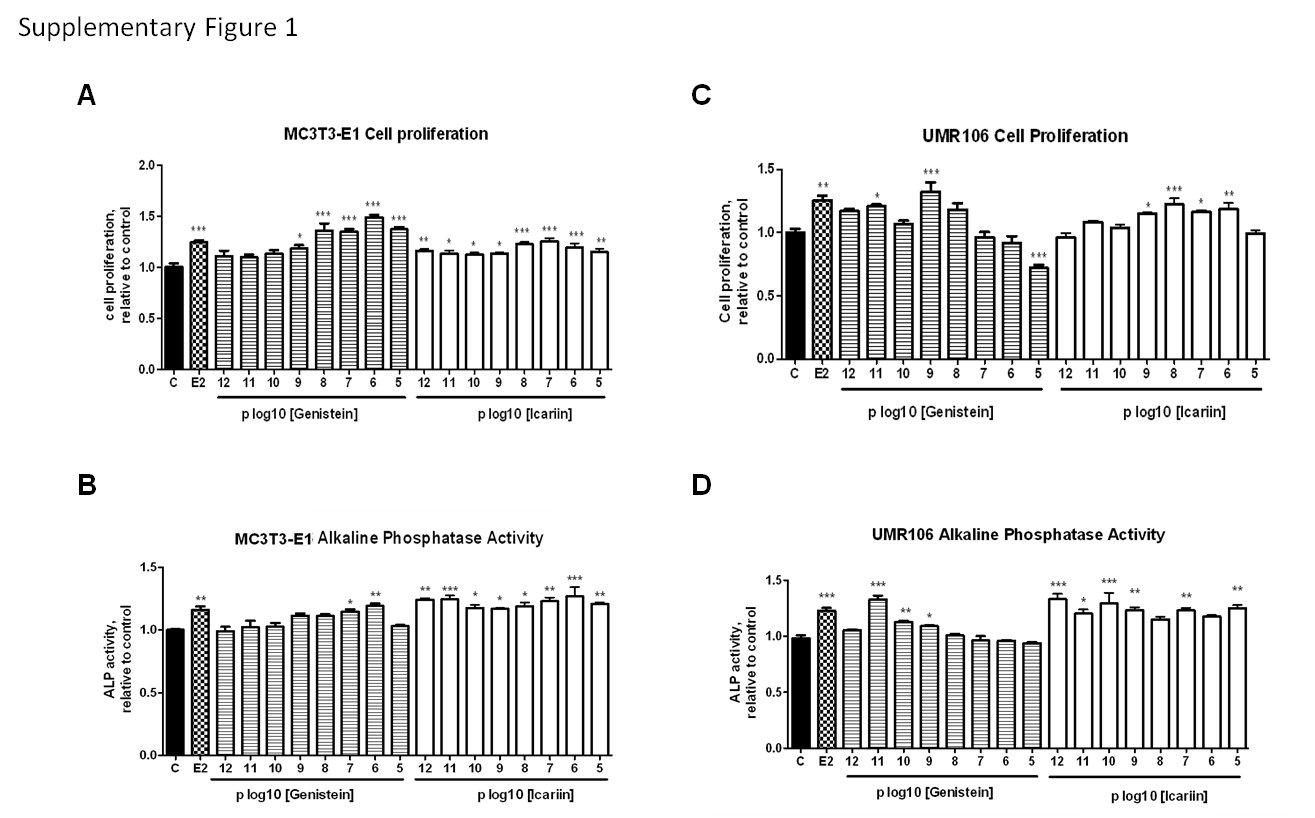

Supplement: FIGURE S1 — The stimulatory effects of genistein and icariin on cell proliferation and differentiation in (A,B) MC3T3-E1 cells and (C,D) UMR-106 cells. Cells were treated with vehicle (C), 17β-estradiol (E2, 10-8M), icariin (10-12–10-5 M), and genistein (10-12–10-5 M) for cell proliferation MTS assay and ALP activity in MC3T3-E1 cells and UMR-106 cells after 48 h and 7 days, respectively. Results were expressed as mean ± SEM. ∗p < 0.05, ∗∗p < 0.01, and ∗∗∗p < 0.001 versus the control (n = 5). [file Image_1.TIF]

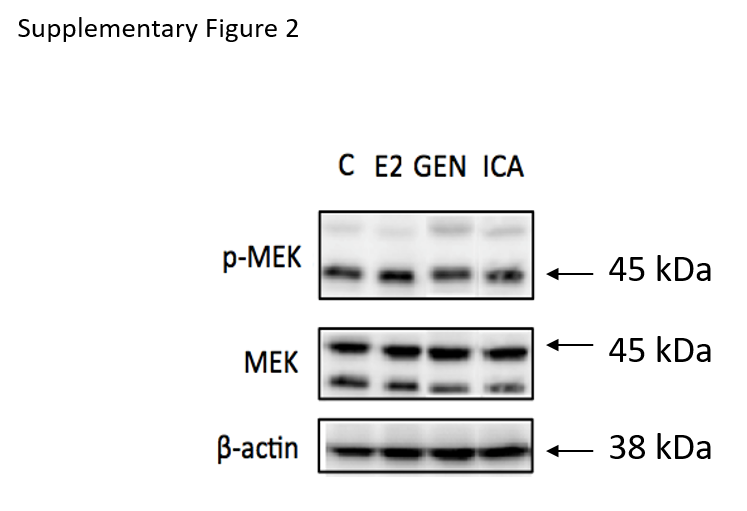

Supplement: FIGURE S2 — Representative immunoblots showing the protein expressions of p-MEK1/2 and MEK1/2 in transfected UMR-106 cells. Cells were treated with vehicle (C), 17β-estradiol (E2, 10-8 M), genistein (GEN; 10 nM), or icariin (ICA; 0.1 μM) for 10 min (n = 3). [file Image_2.TIF]

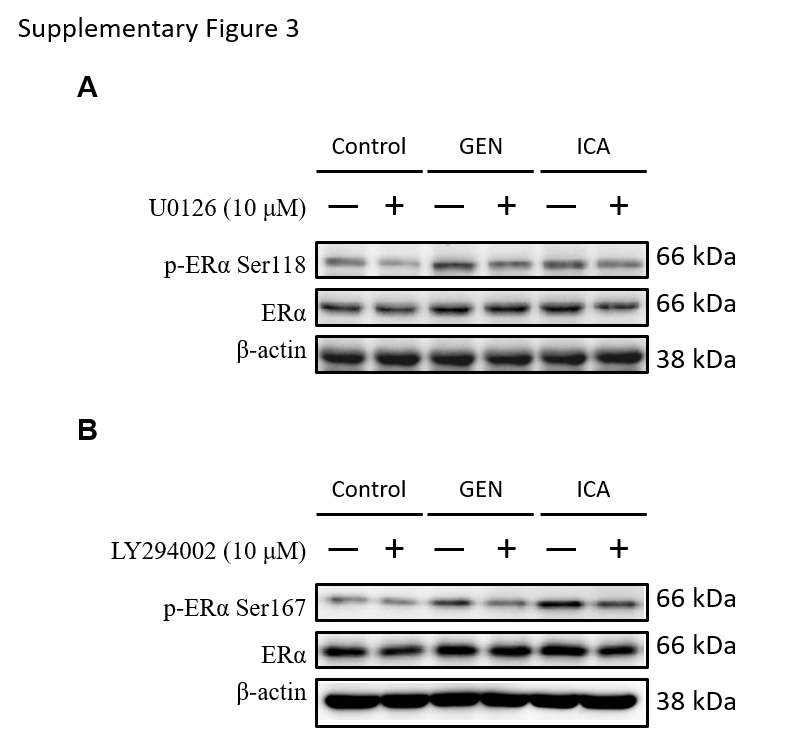

Supplement: FIGURE S3 — The inhibitory effects of the pre-treatment of (A) MAPK inhibitor (U0126) and (B) PI3K inhibitor (LY294002) on flavonoids-induced ERα phosphorylation at Ser118 and Ser167 in osteoblastic MC3T3-E1 cells. Cells were treated with vehicle, genistein (GEN; 10 nM), or icariin (ICA; 0.1 μM) for 10 min after pre-treatment of U0126 (10 μM; 30 min) or LY294002 (10 μM; 30 min). Representative immunoblots showing the protein expressions of p-ERα (Ser118), p-ERα (Ser167), ERα, and β-actin in MC3T3-E1 cells (n = 3). [file Image_3.TIF]
